# Ketamine enhanced ECT in refractory recurrent depression.

**DOI:** 10.1192/j.eurpsy.2024.480

**Published:** 2024-08-27

**Authors:** B. Arribas-Simon, P. Martinez.Gimeno, M. Calvo-Valcarcel, C. Alario-Ruiz, O. Martin-Santiago

**Affiliations:** ^1^Hospital Clinico Universitario, Valladolid, Spain

## Abstract

**Introduction:**

Recurrent Depressive Disorder is a chronic condition that significantly impacts the quality of life. Despite various treatment options, some patients face severe and treatment-resistant relapses. This case is related to research on ketamine in Electroconvulsive Therapy (ECT) for RDD. One study highlighted the efficacy and safety of ketamine compared to other anaesthetic agents in ECT for major depression. Additionally, another study explored subanesthetic doses of ketamine before each ECT session to improve therapeutic outcomes and sleep quality in patients with major depressive disorder.

**Objectives:**

To present a clinical case of a patient with Recurrent Depressive Disorder (RDD) who improved following a change in the Electroconvulsive Therapy (ECT) protocol using ketamine as an anaesthetic inducer.

**Methods:**

We examined the patient’s medical records, including her medical history, previous treatments, and therapeutic responses.

**Results:**

A 65-year-old childless woman with a history of stroke, bilateral carotid atheromatosis, and hypothyroidism suffered from RDD. Despite multiple prior treatments and ECT, she experienced a severe depressive relapse. Eight intensive ECT sessions were administered, with observed memory lapses. Due to the lack of response, the anaesthetic inducer etomidate was replaced with ketamine, resulting in a positive response. The patient continued pharmacological treatment with improved mood, but recent and evident memory alterations persisted, possibly related to anterograde amnesia.

**Conclusions:**

This case highlights the complexity of RDD in patients with comorbidities and treatment-resistant relapses. The change in the ECT protocol using ketamine was effective, emphasizing the importance of alternative therapeutic approaches in refractory cases. The successful treatment of RDD in this patient using ketamine in ECT underscores the need for personalized therapeutic options in treatment-resistant patients. These scientific resources reinforce the relevance of exploring therapeutic alternatives in contemporary clinical practice. We need more research to understand the underlying mechanisms and how this approach could be enhanced in similar cases.

**Disclosure of Interest:**

None Declared

